# Periodontal Disease and Risk of Chronic Obstructive Pulmonary Disease: A Meta-Analysis of Observational Studies

**DOI:** 10.1371/journal.pone.0046508

**Published:** 2012-10-19

**Authors:** Xian-Tao Zeng, Ming-Li Tu, Dong-Yan Liu, Dong Zheng, Jing Zhang, WeiDong Leng

**Affiliations:** 1 Department of Stomatology, Taihe Hospital, Hubei University of Medicne, Shiyan, Hubei Province, People's Republic of China; 2 Department of Respiratory Medicine, Taihe Hospital, Hubei University of Medicne, Shiyan, Hubei Province, People's Republic of China; 3 School of Stomatology, Hubei University of Medicne, Shiyan, Hubei Province, People's Republic of China; The Ohio State Unversity, United States of America

## Abstract

**Background:**

Many epidemiological studies have found a positive association between periodontal disease (PD) and risk of chronic obstructive pulmonary disease (COPD), but this association is varied and even contradictory among studies. We performed a meta-analysis to ascertain the relationship between PD and COPD.

**Methods:**

PubMed and Embase database were searched up to January 10, 2012, for relevant observational studies on the association between PD and risk of COPD. Data from the studies selected were extracted and analyzed independently by two authors. The meta-analysis was performed using the Comprehensive Meta-Analysis software.

**Results:**

Fourteen observational studies (one nested case-control, eight case-control, and five cross-sectional) involving 3,988 COPD patients were yielded. Based on random-effects meta-analysis, a significant association between PD and COPD was identified (odds ratio = 2.08, 95% confidence interval = 1.48–2.91; *P*<0.001), with sensitivity analysis showing that the result was robust. Subgroups analyses according to study design, ethnicity, assessment of PD/COPD, and adjusted/unadjusted odds ratios also revealed a significant association. Publication bias was detected.

**Conclusions:**

Based on current evidence, PD is a significant and independent risk factor of COPD. However, whether a causal relationships exists remains unclear. Morever, we suggest performing randomized controlled trails to explore whether periodontal interventions are beneficial in regulating COPD pathogenesis and progression.

## Introduction

Periodontal Disease (PD) is a group of inflammatory diseases that affecting the supporting tissues of the teeth. At least approximately 35% dentate adults aged between 30 and 90 years old in the United States experience PD [1], and this disease can affect up to 90% of the global population [2]. Based on the theory of “focal infection” which emerged at the beginning of the twentieth century, many studies have investigated a possible role for PD as a risk factor for systemic conditions over the past two decades [3], including cardiovascular diseases [4], diabetes [5], adverse pregnancy outcome [6], osteoporosis [7], rheumatoid arthritis [8], and Chronic obstructive pulmonary disease (COPD) [9], [10].

COPD is the third leading cause of death in the United States, affecting as many as 24 million Americans and resulting in 700,000 hospital admissions, and 124,000 deaths annually [11]. COPD is also an inflammatory disease characterized by the progressive deterioration of pulmonary function and increasing airway obstruction, includes chronic bronchitis and emphysema [12]. In line with the relationship between the anatomical position of oral cavity and pulmonary infection, oral bacteria can be easily carried into the lung and cause infection [13].In addition, PD and COPD share the same risk factors, including smoking, age, obesity, socioeconomic status, and living conditions [3]. These data strongly suggest that PD may be a risk factor for COPD and that oral bacteria may play a key role in its progression.

This hypothesis has received rapidly growing interest in the past years, and the relationship between PD and COPD has been increasingly recognized over the last two decades. Many epidemiological studies [14], [15], [16], [17], [18], [19], [20], [21], [22], [23], [24], [25], [26], [27], [28], [29], [30], [31], [32], [33], [34] have investigated the link between PD and risk of COPD, and most found a positive association. However, different studies have used different measurement methods and investigated different populations, and the magnitudes of the said association are varied and even contradictory among studies. Moreover, the possible role of PD in the pathogenesis of COPD remains an important but unresolved issue.

Two previous systematic reviews [9], [10] combined several epidemiological studies and reported an association between respiratory diseases and oral health, involving PD and COPD. One review [9] reported a weak association between PD and COPD based on two case-control studies [14], [16] and two cross-sectional studies [15], [34] of poor to fair quality, whereas the other [10] demonstrated a potential association between PD and COPD based on one case-control studies [14] and three cross-sectional studies [15], [18], [34]. The evidence from each work is limited because only four studies were available and the conventional risk factors were not adjusted in the analysis. The presence of common conventional risk factors, such as smoking, renders the results questionable. Furthermore, whether PD is an independent risk factor for or merely a silent marker of COPD remains unclear.

An improved understanding of this issue may have important public health and clinical implications given the possibility that prevention and treatment of PD might reduce the incidence of COPD. The objectives of this study were to (1) evaluate the inconsistent results from published observational studies on the association between PD and risk of COPD by conducting a meta-analysis and (2) gain a more robust estimate association. We followed the PRISMA (Preferred Reporting Items for Systematic Reviews and Meta-Analyses) [35] guidelines to report this meta-analysis.

## Methods

### Literature search

We initially identified published studies that investigated the association between PD and risk of COPD by searching the PubMed and Embase databases from their inception through to January 10, 2012. The following search terms were used: (1) “periodontal disease” or “paradontosis” or “parodontopathy” or “periodontal” or “gum disease” or “periodontium”, and (2) “chronic obstructive pulmonary diseases” or “COPD” or “chronic bronchitis” or “emphysema”, without restrictions. We also reviewed the reference lists of retrieved articles and recent reviews.

### Study selection

We included any study that met all of the following criteria: (1) the study was of a cross-sectional, case-control, or cohort design; (2) clear diagnostic criteria for PD and COPD were established; (3) the association between PD and risk of COPD was investigated; and (4) the odds ratios (ORs)/risk ratio (RR, for cohort studies) and the corresponding 95% confidence intervals (CIs), or the number of events that can calculate them were reported. Two authors independently evaluated the eligibility of all studies retrieved from the databases. Disagreements were resolved by discussion or in consultation with a third author.

### Data extraction

Two reviewers independently extracted data about the characteristics of each study by using a standardized data collection form. Data were recorded as follows: first author's last name, year of publication, country of origin; characteristics of study population and age at baseline; number of participants with PD and total number of participants, ascertainment of PD; assessment of COPD; and statistical adjustments for confounding factors. Any disagreements were resolved by consensus.

### Statistical analysis

We proposed that results from single studies be pooled by meta-analysis where this was found to be both clinically and statistically appropriate. We computed pooled ORs/RRs and relevant 95% CIs using Comprehensive Meta-Analysis software, Version 2.2 (Biostat, Englewood, NJ, USA) [36] to generate forest plots, determine whether a statistical association between PD and risk of COPD exists, and assess the heterogeneity of the selected studies. Heterogeneity was quantified using the *I* ^2^ statistic [37], with the low, moderate, and high to *I* ^2^ values of 25%, 50%, and 75% respectively [38]. Where the *I* ^2^ value was 25% or lower, indicating no evidence of heterogeneity, we used the fixed-effect model; otherwise, we used the random-effects model.

In the presence of heterogeneity, we performed subgroup and sensitivity analyses to explore possible explanations for the heterogeneity and examine the influence of various exclusion criteria on the overall risk estimate. We also investigated the influence of a single study on the overall risk estimate by sequentially removing study to test the robustness of the main results.

Where possible, potential publication bias was assessed by visual inspection of the funnel plots of the primary outcome. The Egger linear regression test was used to examine the association between mean effect estimate and its variance [39]. In addition, to assess the effect of possible publication bias, we calculated the number of unpublished studies that would have to exist to negate the results and the pooled OR adjusted for publication bias using the trim and fill method [40].

## Results

### Study identification

**Figure 1:**
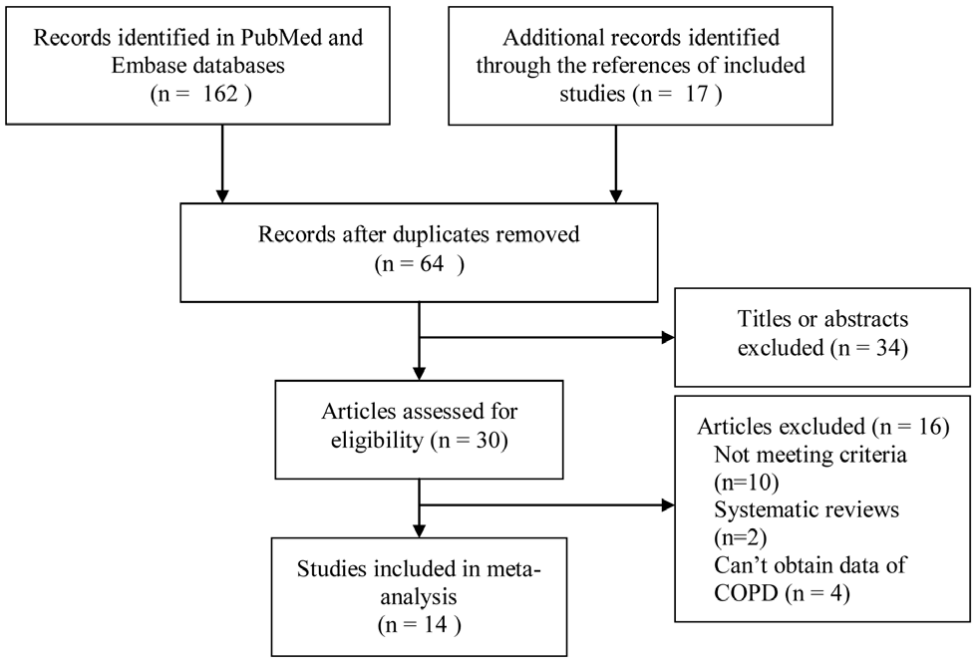
Flow chart from identification of studies that tested the association between PD and risk of COPD to final inclusion. PD, periodontal dsease; COPD, chronic obstructive pulmonary disease.

Of 179 records found initially, 14 articles involving 1 nested case-control study [14], 8 case-control studies [16], [20], [22], [23], [24], [25], [26], [27], and 5 cross-sectional studies [15], [18], [19], [21], [34] were included in this meta-analysis. A detailed flowchart of the selection process is shown in Figure 1.

### Study characteristics

Table 1 presents the major characteristics of the 14 studies for which information was available. These studies focused on COPD only. Sample sizes ranged from 58 to 13,792, representing 3,988 COPD patients and 22,871 controls subjects. One study provided results for males only [14], whereas another did for males in the COPD group and both sexes in the control group [25]; data for 2 studies [18], [23], and 10 others reported results for both sexes [15], [16], [19], [20], [21], [22], [24], [26], [27], [34]. Of the principal studies, 6, 2, and 3 were conducted in the United States [14], [15], [16], [18], [19], [34], India [22], [25], and China [24], [26], [27], respectively; 1 study each was conducted in Poland [20], Norway [21], and Iran [23].

**Table 1:** Study characteristics of included studies.

Refence	Design	Location	Group	Subject	Age(yrs)	Gender(♂/♀)	Assessment of PD	Assessment of COPD	Adjustment for Covariates	OR(95%CI)
Hayes et al 1998	NCC	USA	COPD	261	45.06±9.7	261/0	ABL	FEV1	age, smoking, education, and height	1.77(1,27,2.48)
			Control	857	42.18±9.1	857/0				
Scannapieco et al 1998	CS	USA	COPD	77	NA	45/32	OHI	Self-reported	NA	4.5(1.06,18.99)
			Control	309		137/172				
Russell et al 1999	CC	USA	COPD	28	75.9±8.1	14/14	PI	Self-reported	NA	15(1.59,130.23)
			Control	30	75.1±5.9	12/18				
Scannapieco et al 2001	CS	USA	COPD	810	51.2±17.9	304/506	CAL	FEV1/FVC	age, gender, race and ethnicity, and education, income, number of dental visits, pack years of smoking, alcohol consumption, and diabetes mellitus	1.45(1.02,2.05)
			Control	12,982	43.9±17.7	6,161/6,821				
Garcia et al 2001	CS	USA	COPD	279	NA	NA	ABL	FEV1	age, height, smoking, alcohol, and education	1.75(1.33,2.30)
			Control	833						
Hyman et al 2004	CS	USA	COPD	993	62.32±14.07	3,636/2,296	CAL	GOLD criteria	age, gender, race/ethnicity,history of hypertension, history of heart attack, dental visit within 1 year, body mass index, smoking, and family income	1.48(0.90,2.43)
			Control	6,632	47.37±14.23	371/662				
Kowalski et al 2005	CC	Poland	COPD	100	63.1±10.17	68/32	PI	FEV1/FVC	NA	3.59(1.84,6.98)
			Control	101	65.3±10.36	38/63				
Leuckfeld 2008	CS	Norway	COPD	130	54.9±4.9	50/80	ABL	GOLD criteria	age, gender, and smoking	10.0(1.03,97.47)
			Control	50	47.0±9.8	29/21				
Fatemi et al 2009	CC	Iran	COPD	30	53±7	NA	CAL	PFT	NA	1.80(1.46,2.22)
			Control	30	54±5					
Wang 2009	CC	China	COPD	306	63.94±9.84	210/96	CAL	GOLD criteria	age, gender, smoking, and body mass index	1.00(0.99,1.01)
			Control	328	63.26±8.98	164/164				
Deo et al 2009	CC	India	COPD	150	41.43±7.53	140/10	CAL	FEV1/FVC	NA	1.11(0.79,1.56)
			Control	50	43.625.53	38/12				
Prasanna 2011	CC	India	COPD	50	56.3±3.8	50/0	PI	PFT	NA	3.12(2.53,3.85)
			Control	50	47.4±4.9	27/23				
Si et al 2012	CC	China	COPD	581	63.9±9.4	422/159	PI	FEV1/FVC	age, gender, occupation, educational level and smoking status	9.01(3.98,20.40)
			Control	438	62.8±9.5	218/220				
Zhou et al 2012	CC	China	COPD	193	63.6±10.3	132/61	ABL	FEV1/FVC	age, gender, smoking, and body mass index	1.34(0.66,2.73)
			Control	181	62.1±9.06	108/73				

NCC, nest case-control study; CS, cross-sectional study; CC, case-control study; NA, not available; ABL, alveolar bone loss; OHI, oral health index; PI, periodontal index; CAL, clinical attachment loss; FEV1, forced expiratory volume in 1 second; FVC, forced vital capacity; GOLD criteria, Global Initiative for Chronic Obstructive Lung Disease spirometry guidelines; PFT, pulmonary function test, that means the article just mentioned but did not give what measurement have been used.

### PD and risk of COPD

**Figure 2:**
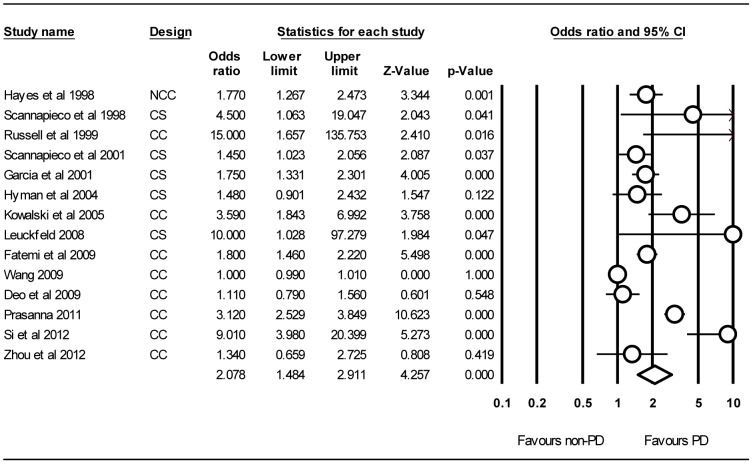
Forest plot of PD and risk of COPD, studies are pooled with random-effects model. The pooled OR was represented by a diamond of standard height, with the width indicating the 95% CI. PD, periodontal dsease; COPD, chronic obstructive pulmonary disease; OR, odds ratio; CI, confidence interval; NCC, nest case-control study; CC, case-control study; CS, cross-sectional study.

Figure 2 shows the results from the random-effects model pooling the ORs and 95% CIs for COPD. Of all 14 studies, four studies show there are no statistical differences [19], [22], [24], [27]; six studies show that population with PD face more than double risk of developing COPD compare with without ones [15], [16], [20], [21], [25], [26], and two studies show this times are ten or more [16], [21]. The overall meta-analysis of 14 studies identified a significantly increased risk of developing COPD (OR = 2.08, 95% CI = 1.48–2.91; *P*<0.001). Substantial heterogeneity was observed (*I* ^2^ = 94.4%, *P*<0.001).

### Subgroup and sensitivity analyses

**Figure 3:**
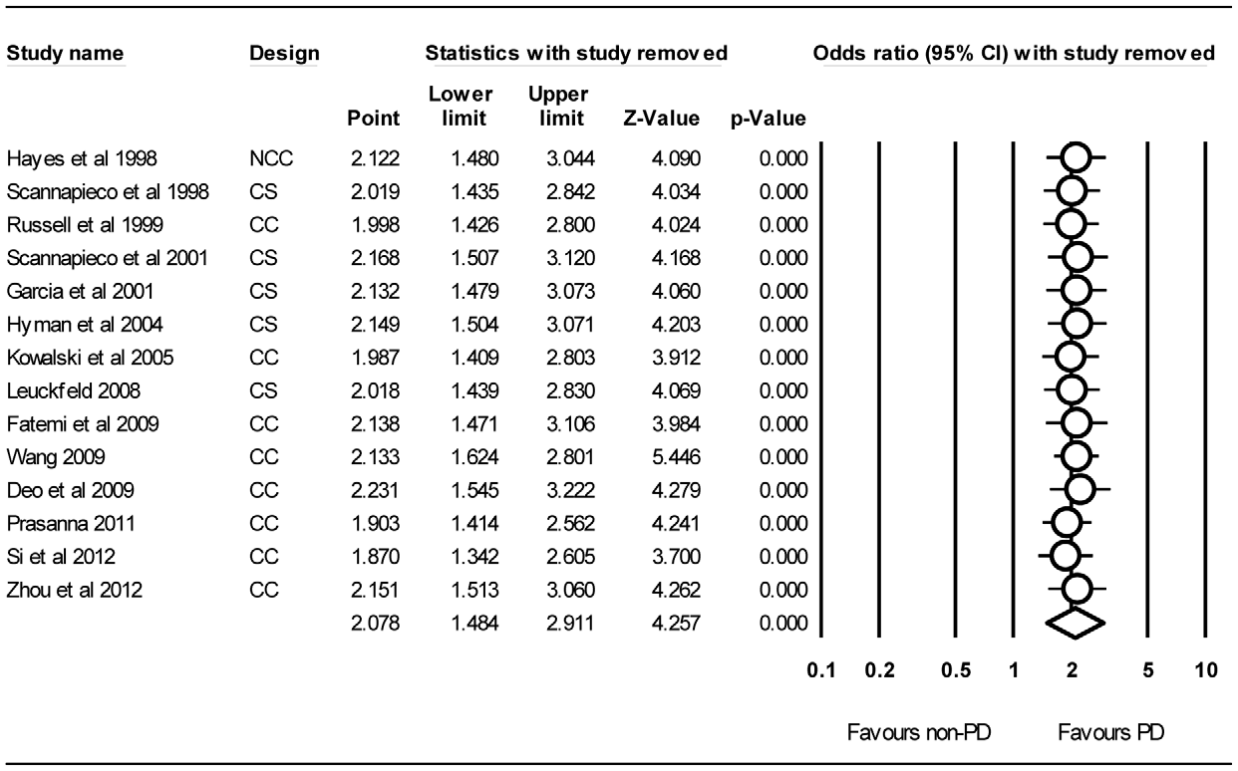
Forst plot of sensitivity analysis by removing each study in each turn. The pooled OR was represented by a diamond of standard height, with the width indicating the 95% CI. PD, periodontal dsease; COPD, chronic obstructive pulmonary disease; OR, odds ratio; CI, confidence interval; NCC, nest case-control study; CC, case-control study; CS, cross-sectional study.

Table 2 shows the results of subgroup analyses by study design, ethnicity, assessment of PD, assessment of COPD, and adjustment for covariates. All these analyses indicated that PD is a risk factor for developing COPD, except for the clinical attachment loss (CAL) (OR = 1.32, 95% CI = 0.97–1.80; *P* = 0.07) and GOLD criteria (OR = 1.31, 95% CI = 0.76–2.24; *P* = 0.33). When stratified by adjustment for covariates, all the eight studies have adjusted smoking [14], [18], [19], [21], [24], [26], [27], [34], and the risk of adjusted studies is lower (OR = 1.78, 95% CI = 1.23–2.58; *P*<0.001) than unadjusted ones (OR = 2.40, 95% CI = 1.52–3.81; *P*<0.001), and the relevant 95%CI is also narrower. Sensitivity analysis was performed by sequentially removing each study. The significance of pooled ORs was not greatly influenced by the omission of any single study (the values of ORs are between 1.87 and 2.23, and the relevant 95%CIs are between 1.34 and 3.22), suggesting that the results of this meta-analysis are stable (Fig. 3).

**Table 2:** Results of subgroups analyses of pooled ORs (95% CIs).

Subgroups	Num. of studies	Meta-analyses			Effect model	Heterogeneity	
		ORs	95% CIs	p value		I^2^	p value
Study design							
NCC	1	2.77	1.27-2.47	<0.001	fixed	0	1.00
CC	8	2.26	1.38-3.70	<0.001	random	96.33%	<0.001
CS	5	1.35	1.66-1.36	<0.001	fixed	22.38%	0.27
Ethnicity							
North America	6	1.69	1.43-2.00	<0.001	fixed	25.30%	0.24
Asia	6	1.93	1.15-3.26	<0.001	random	97.08%	<0.001
Europe	2	3.89	2.05-7.38	<0.001	fixed	0	0.40
Assessment of PD							
ABL	4	1.74	1.42-2.13	<0.001	fixed	0	0.42
CAL	5	1.32	0.97-1.80	0.07	random	89.25%	<0.001
OHI	1	4.50	1.06-19.05	0.05	fixed	0	1.00
PI	4	4.55	2.59-7.98	<0.001	random	61.92%	0.05
Assessment of COPD							
FEV1	2	1.76	1.42-2.17	<0.001	fixed	0	0.96
FEV1/FEC	5	2.18	1.19-4.01	<0.001	random	85.62%	<0.001
GOLD criteria	3	1.31	0.76-2.24	0.33	random	68.39%	0.04
Self-reported	2	6.46	1.93-21.60	<0.001	fixed	0	0.37
PFT	2	2.37	1.38-4.06	<0.001	random	92.43%	<0.001
Adjustment for covariates							
Yes	8	1.78	1.23-2.58	<0.001	random	89.42%	<0.001
NA	6	2.40	1.52-3.81	<0.001	random	85.95%	<0.001

NCC, nest case-control study; CS, cross-sectional study; CC, case-control study; NA, not available; ABL, alveolar bone loss; OHI, oral health index; PI, periodontal index; CAL, clinical attachment loss; FEV1, forced expiratory volume in 1 second; FVC, forced vital capacity; GOLD criteria, Global Initiative for Chronic Obstructive Lung Disease spirometry guidelines; PFT, pulmonary function test, that means the article just mentioned but did not give what measurement have been used.

### Publication bias

**Figure 4:**
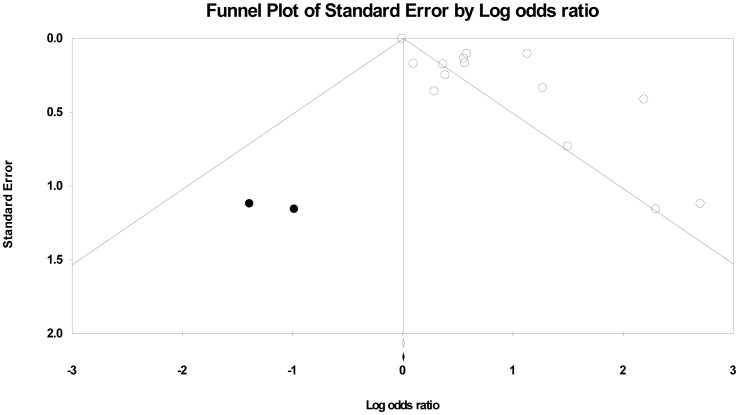
Filled funnel plot with pseudo-95% CIs of results of 14 studies. Log of OR represents the natural logarithm of the OR of individual studies; Standard Error by Log odds ratio represents the standard error in the natural logarithm of the OR of individual studies. A circle in the figure represents a study, while a black spot represents an unpublished study that would have to exist to negate the results of the meta-analysis.

Figure 4 shows that the funnel plot was asymmetrical, indicating the presence of publication bias. The Egger linear regression test also detected moderate publication bias among studies (OR = 3.43, 95%CI = 1.76–5.10; *P*<0.001). As the evidence of bias could be due to inadequate statistical power, we used the non-parametric method of “trim and fill” and estimated two possible missing studies (black spots in Fig. 4). The estimated OR including the “missing” studies did not substantially different from our estimate with adjustment for missing studies (OR = 1.94, 95%CI = 1.40–2.70).

## Discussion

COPD includes chronic bronchitis and emphysema, Murray et al has predicted that COPD would become the third most common cause of death and the fourth most important disease leading to disability by 2020 [41], [42]. PD is considered a novel risk factor, in addition to smoking, chronic exposure to hazardous air pollutants, and genetic conditions [43]. Our meta-analysis of one nested case-control study, eight case-control studies, and five cross-sectional studies provides evidence that PD is associated with an increased risk of COPD and can increase a significantly the risk by 2.08 times. When the subjects were stratified by ethnicity, this association did not differ: the ORs were 1.69 for North Americans (95%CI = 1.43–2.00), 1.93 for Asians (95%CI = 1.15–3.26), and 3.89 for Europeans (95%CI = 2.05–7.38). When the analysis was restricted to studies with control for conventional rsik factors, including age, gender, and especially smoking, PD was identified as a probable independent risk factor for COPD (OR = 1.78, 95%CI = 1.23–2.58; *P*<0.001). When the groups were stratified the group by study design, the results also suggested that PD is a significant risk factor for COPD in both case-control and cross-sectional studies, with the association gaining more powerful from the cross-sectional studies (OR = 1.35, 95%CI = 1.66–1.36; *P*<0.001) to the case-control studies (OR = 2.26, 95%CI = 1.38–3.70; *P*<0.001) to the nested case-control study (OR = 2.77, 95%CI = 1.27–2.47; *P*<0.001). This finding can be attributed to the fact that the cross-sectional study design suffers from more confounding biases compared with the case–control study design and the case–control study design suffers from more confounding biases compared with the nested case–control study design. The prospective cohort study design is the best among observational studies, and it can control confounding biases very well. We therefore suggest that this study design be used in future research.

Oral cavity has been recognized as a potential reservoir for respiratory pathogens [32], and dental plaque may play an important role in its formation, which may also contribute to PD [44]. Colonization of dental plaque by respiratory pathogens followed by aspiration has been proposed as a possible mechanism, but data on whether the same pathogens are isolated in PD and COPD exacerbations are limited. Epidemiological studies have found evidence favoring this association; however, debate on this issue has ensued over recent decades. Our meta-analysis detected a significant association between PD and COPD with and without adjustments made on the conventional risk factors. This suggests that an effective oral hygiene regimen would effectively prevent the progression of COPD, and that an effective PD intervention treatment should be able to control COPD. Correct and effective brushing of teeth, use of dental floss, and regular periodontal scaling would be the simplest and most cost-effective actions. To substantiate these findings, we recommend that sufficient relevant randomized controlled trials be carried out to test whether periodontal interventions can affect the progression of COPD. Moreover, we suggest that fundamental studies investigating the true mechanism between PD and COPD be performed, particularly to identify which role or roles would take much of the responsibility and whether the pathogens isolated in PD exacerbations are the same as those isolated in COPD exacerbations — which can give clearer guidelines for clinical practice and make the link between the two diseases more convincing. In addition, a spacer device and cold water for rinsing should be used in COPD patients undergoing treatment that requires steroid inhalation to reduce the accumulation of steroids in the mouth cavity; similarly, when a metered-dose inhaler is used for steroid inhalation, a spacer device should be applied to reduce the possibility for respiratory pathogens in dental plaque to move into the lung.

The main strength of this study was pooling data from individual studies with small sample sizes and a low statistical power via meta-analysis approach. We also collected studies as relevant as possible and used subgroup and sensitivity analyses to test the robustness of the results. This meta-analysis overcame the shortcomings of two pervious systematic reviews (our meta-analysis also included the five studies [14], [15], [16], [18], [34] that used in those systematic reviews) [9], [10].

However, this study also had some limitations. First, although meta-analysis is a useful tool in epidemiology, problems associated with methodology may limit its benefits. Case-control study and cross-sectional studies are not the best designs among observational studies; thus, evidence from these studies is likely to be less accurate and possibly more influenced by recall bias compared with that from cohort studies. In addition, meta-analysis is a secondary analysis approach and has some inevitable biases. Second, some pooled ORs were obtained from heterogeneous studies, although we performed sensitivity analyses. Third, the funnel plot of publication bias showed that it was asymmetrical, and publication bias thus had to be considered. This suggests that we may have missed important unpublished studies with results inconsistent with our findings, although we did our best to collect all relevant studies. The filled funnel plot showed that two additional unpublished studiesare needed to negate the results of our meta-analysis. Fourth, none of the 14 studies were selected in our meta-analysis provided the degrees of PD and risk of COPD such that we could not conduct a dose-response analysis to assess their relationship more precisely. Fifth, the prevalence/incidence of COPD in developing countries (where access to dental care is limited) is presumably much higher than that in developed countries (where access to dental care is better), but we could not obtain current and relevant data to verify this. As PD and COPD share many risk factors, further research should analyze whether the association between them is causal using validated and optimal measurement tools (eg., Community Periodontal Index of Treatment Needs [45] for PD and FEV1/FVC for COPD [46]), calculate the interval between the onset of each disease, and consider adequate control for the confounding factors.

## Conclusions

Based on current evidences, it can be concluded that: (1) PD significantly increases the risk of COPD, with the increase being likely independent of conventional COPD risk factors. However, the mechanisms still not clear. We suggest performing fundamental studies to investigate the true mechanism between PD and COPD. (2) Dental plaque that contains bacteria may be responsible for COPD, therefore, good attention to teeth brushing and general oral hygiene care may reduce the risk of COPD, and further studies are recommended to elucidate whether periodontal interventions can control the progression or reduce the incidence of COPD. (3) When using inhaled steroids treatment, COPD patients are suggested to use a spacer and rinse mouth with cold water. (4) An individual who is susceptible to COPD should pay close attention to his/her oral hygiene. It is suggested, for example, that the client brush twice a day with an antibacterial dentifrice, arrange for regular dental check-ups, and refrain from smoking. (5) An individual suffering from PD should seek treatment immediately and adhere to the doctor's orders concerning said treatment. (6) Those using removable dentures should clean them daily with/by polident, and those with permanent dentures should be examined at least every two years.

## References

[1] Albandar JM, Brunelle JA, Kingman A (1999) Destructive periodontal disease in adults 30 years of age and older in the United States, 1988–1994. J Periodontol 70: 13–29. 10052767 10.1902/jop.1999.70.1.13

[2] Pihlstrom BL, Michalowicz BS, Johnson NW (2005) Periodontal diseases. Lancet 366: 1809–1820. 16298220 10.1016/S0140-6736(05)67728-8

[3] Cullinan MP, Ford PJ, Seymour GJ (2009) Periodontal disease and systemic health: current status. Aust Dent J 54 Suppl 1: S62–69. 19737269 10.1111/j.1834-7819.2009.01144.x

[4] Blaizot A, Vergnes JN, Nuwwareh S, Amar J, Sixou M (2009) Periodontal diseases and cardiovascular events: meta-analysis of observational studies. Int Dent J 59: 197–209. 19774803

[5] Preshaw PM, Alba AL, Herrera D, Jepsen S, Konstantinidis A, et al (2012) Periodontitis and diabetes: a two-way relationship. Diabetologia 55: 21–31. 22057194 10.1007/s00125-011-2342-y PMC3228943

[6] Xiong X, Buekens P, Vastardis S, Yu SM (2007) Periodontal disease and pregnancy outcomes: state-of-the-science. Obstet Gynecol Surv 62: 605–615. 17705886 10.1097/01.ogx.0000279292.63435.40

[7] Megson E, Kapellas K, Bartold PM (2010) Relationship between periodontal disease and osteoporosis. Int J Evid Based Healthc 8: 129–139. 21199381 10.1111/j.1744-1609.2010.00171.x

[8] Detert J, Pischon N, Burmester GR, Buttgereit F (2010) The association between rheumatoid arthritis and periodontal disease. Arthritis Res Ther 12: 218. 21062513 10.1186/ar3106 PMC2990988

[9] Azarpazhooh A, Leake JL (2006) Systematic review of the association between respiratory diseases and oral health. J Periodontol 77: 1465–1482. 16945022 10.1902/jop.2006.060010

[10] Scannapieco FA, Bush RB, Paju S (2003) Associations between periodontal disease and risk for nosocomial bacterial pneumonia and chronic obstructive pulmonary disease. A systematic review. Ann Periodontol 8: 54–69. 14971248 10.1902/annals.2003.8.1.54

[11] Corbridge S, Wilken L, Kapella MC, Gronkiewicz C (2012) An evidence-based approach to COPD: part 1. Am J Nurs 112: 46–57; quiz 59,58. 22333971 10.1097/01.NAJ.0000412639.08764.21

[12] Hill K, Goldstein RS, Guyatt GH, Blouin M, Tan WC, et al (2010) Prevalence and underdiagnosis of chronic obstructive pulmonary disease among patients at risk in primary care. CMAJ 182: 673–678. 20371646 10.1503/cmaj.091784 PMC2855915

[13] Gomes-Filho IS, Passos JS, Seixas da Cruz S (2010) Respiratory disease and the role of oral bacteria. J Oral Microbiol 2. 10.3402/jom.v2i0.5811 PMC3084574 21523216

[14] Hayes C, Sparrow D, Cohen M, Vokonas PS, Garcia RI (1998) The association between alveolar bone loss and pulmonary function: the VA Dental Longitudinal Study. Ann Periodontol 3: 257–261. 9722709 10.1902/annals.1998.3.1.257

[15] Scannapieco FA, Papandonatos GD, Dunford RG (1998) Associations between oral conditions and respiratory disease in a national sample survey population. Ann Periodontol 3: 251–256. 9722708 10.1902/annals.1998.3.1.251

[16] Russell SL, Boylan RJ, Kaslick RS, Scannapieco FA, Katz RV (1999) Respiratory pathogen colonization of the dental plaque of institutionalized elders. Spec Care Dentist 19: 128–134. 10860077 10.1111/j.1754-4505.1999.tb01413.x

[17] Scannapieco FA (1999) Role of oral bacteria in respiratory infection. Journal of Periodontology 70: 793–802. 10440642 10.1902/jop.1999.70.7.793

[18] Garcia RI, Nunn ME, Vokonas PS (2001) Epidemiologic associations between periodontal disease and chronic obstructive pulmonary disease. Ann Periodontol 6: 71–77. 11887473 10.1902/annals.2001.6.1.71

[19] Hyman JJ, Reid BC (2004) Cigarette smoking, periodontal disease, and chronic obstructive pulmonary disease. Journal of Periodontology 75: 9–15. 15025211 10.1902/jop.2004.75.1.9

[20] Kowalski M, Kowalska E, Split M, Split W, Wierzbicka-Ferszt A, et al (2005) Assessment of periodontal state in patients with chronic obstructive pulmonary disease - Part II. Polski Merkuriusz Lekarski 19: 537–541. 16379320

[21] Leuckfeld I, Obregon-Whittle MV, Lund MB, Geiran O, Bjortuft O, et al (2008) Severe chronic obstructive pulmonary disease: Association with marginal bone loss in periodontitis. Respiratory Medicine 102: 488–494. 18191392 10.1016/j.rmed.2007.12.001

[22] Deo V, Bhongade ML, Ansari S, Chavan RS (2009) Periodontitis as a potential risk factor for chronic obstructive pulmonary disease: a retrospective study. Indian J Dent Res 20: 466–470. 20139573 10.4103/0970-9290.59456

[23] Fatemi K, Banihashemrad S, Tovhidi M, Hosseini S (2009) Evaluation of the Relationship Between Periodontal Disease and Chronic Obstructive Pulmonary Disease. J Mash Dent Sch 33: 214–216.

[24] Wang Z, Zhou X, Zhang J, Zhang L, Song Y, et al (2009) Periodontal health, oral health behaviours, and chronic obstructive pulmonary disease. J Clin Periodontol 36: 750–755. 19614723 10.1111/j.1600-051X.2009.01448.x

[25] Prasanna SJ (2011) Causal relationship between periodontitis and chronic obstructive pulmonary disease. J Indian Soc Periodontol 15: 359–365. 22368360 10.4103/0972-124X.92570 PMC3283933

[26] Si Y, Fan H, Song Y, Zhou X, Zhang J, et al (2012) Association Between Periodontitis and Chronic Obstructive Pulmonary Disease (COPD) in a Chinese Population. J Periodontol 10.1902/jop.2012.110472 22248220

[27] Zhou X, Han J, Song Y, Zhang J, Wang Z (2012) Serum levels of 25-hydroxyvitamin D, oral health and chronic obstructive pulmonary disease. J Clin Periodontol 39: 350–356. 22296704 10.1111/j.1600-051X.2012.01852.x

[28] Katancik JA, Kritchevsky S, Weyant RJ, Corby P, Bretz W, et al (2005) Periodontitis and airway obstruction. Journal of Periodontology 76: 2161–2167. 16277589 10.1902/jop.2005.76.11-S.2161

[29] Terpenning MS, Taylor GW, Lopatin DE, Kerr CK, Dominguez BL, et al (2001) Aspiration pneumonia: dental and oral risk factors in an older veteran population. J Am Geriatr Soc 49: 557–563. 11380747 10.1046/j.1532-5415.2001.49113.x

[30] Terpenning MS (2001) The relationship between infections and chronic respiratory diseases: an overview. Annals of periodontology/the American Academy of Periodontology 6: 66–70. 10.1902/annals.2001.6.1.66 11887472

[31] Sharma N, Shamsuddin H (2011) Association between respiratory disease in hospitalized patients and periodontal disease: A cross-sectional study. Journal of Periodontology 82: 1155–1160. 21219100 10.1902/jop.2011.100582

[32] Mojon P (2002) Oral health and respiratory infection. J Can Dent Assoc 68: 340–345. 12034069

[33] Liu Z, Zhang W, Zhang J, Zhou X, Zhang L, et al (2012) Oral hygiene, periodontal health and chronic obstructive pulmonary disease exacerbations. J Clin Periodontol 39: 45–52. 22092913 10.1111/j.1600-051X.2011.01808.x

[34] Scannapieco FA, Ho AW (2001) Potential associations between chronic respiratory disease and periodontal disease: analysis of National Health and Nutrition Examination Survey III. J Periodontol 72: 50–56. 11210073 10.1902/jop.2001.72.1.50

[35] Moher D, Liberati A, Tetzlaff J, Altman DG, Group P (2009) Preferred reporting items for systematic reviews and meta-analyses: the PRISMA statement. BMJ 339: b2535. 19622551 10.1136/bmj.b2535 PMC2714657

[36] Borenstein M, Hedges L, Rothstein H (2005) Comprehensive Meta-analysis. Version 2 ed. Biostat, Englewood, New Jersey.

[37] Higgins JP, Thompson SG (2002) Quantifying heterogeneity in a meta-analysis. Stat Med 21: 1539–1558. 12111919 10.1002/sim.1186

[38] Higgins JP, Thompson SG, Deeks JJ, Altman DG (2003) Measuring inconsistency in meta-analyses. BMJ 327: 557–560. 12958120 10.1136/bmj.327.7414.557 PMC192859

[39] Egger M, Davey Smith G, Schneider M, Minder C (1997) Bias in meta-analysis detected by a simple, graphical test. BMJ 315: 629–634. 9310563 10.1136/bmj.315.7109.629 PMC2127453

[40] Duval S, Tweedie R (2000) Trim and fill: A simple funnel-plot-based method of testing and adjusting for publication bias in meta-analysis. Biometrics 56: 455–463. 10877304 10.1111/j.0006-341x.2000.00455.x

[41] Murray CJ, Lopez AD (1997) Alternative projections of mortality and disability by cause 1990–2020: Global Burden of Disease Study. Lancet 349: 1498–1504. 9167458 10.1016/S0140-6736(96)07492-2

[42] Murray CJ, Lopez AD (1997) Global mortality, disability, and the contribution of risk factors: Global Burden of Disease Study. Lancet 349: 1436–1442. 9164317 10.1016/S0140-6736(96)07495-8

[43] Scannapieco FA (1999) Role of oral bacteria in respiratory infection. J Periodontol 70: 793–802. 10440642 10.1902/jop.1999.70.7.793

[44] Coulthwaite L, Verran J (2007) Potential pathogenic aspects of denture plaque. Br J Biomed Sci 64: 180–189. 18236742 10.1080/09674845.2007.11732784

[45] Croxson LJ (1984) A simplified periodontal screening examination: the Community Periodontal Index of Treatment Needs (WHO) in general practice. Int Dent J 34: 28–34. 6584398

[46] McKusick KA, Wagner HN Jr, Soin JS, Benjamin JJ, Cooper M, et al (1974) Measurement of regional lung function in the early detection of chronic obstructive pulmonary disease. Scand J Respir Dis Suppl 85: 51–63. 4534922

